# Golgi-associated LC3 lipidation requires V-ATPase in noncanonical autophagy

**DOI:** 10.1038/cddis.2016.236

**Published:** 2016-08-11

**Authors:** Ying Gao, Yajun Liu, Liang Hong, Zuolong Yang, Xinran Cai, Xiaoyun Chen, Yuanyuan Fu, Yujie Lin, Weijie Wen, Sitong Li, Xingguo Liu, Heqing Huang, Andreas Vogt, Peiqing Liu, Xiao-Ming Yin, Min Li

**Affiliations:** 1 National and Local United Engineering Lab of Druggability and New Drugs Evaluation, School of Pharmaceutical Sciences, Sun Yat-Sen University, Guangzhou 510006, China; 2 Department of Pathology and Laboratory Medicine, Indiana University School of Medicine, Indianapolis, IN 46202, USA; 3 Guangzhou Institute of Biomedicine and Health, Chinese Academy of Sciences, Guangzhou 510530, China; 4 Department of Computational and Systems Biology, University of Pittsburgh School of Medicine, Pittsburgh, PA 15261, USA

## Abstract

Autophagy is an evolutionarily conserved catabolic process by which cells degrade intracellular proteins and organelles in the lysosomes. Canonical autophagy requires all autophagy proteins (ATGs), whereas noncanonical autophagy is activated by diverse agents in which some of the essential autophagy proteins are dispensable. How noncanonical autophagy is induced and/or inhibited is still largely unclear. In this study, we demonstrated that AMDE-1, a recently identified chemical that can induce canonical autophagy, was able to elicit noncanonical autophagy that is independent of the ULK1 (unc-51-like kinase 1) complex and the Beclin1 complex. AMDE-1-induced noncanonical autophagy could be specifically suppressed by various V-ATPase (vacuolar-type H^+^-ATPase) inhibitors, but not by disturbance of the lysosome function or the intracellular ion redistribution. Similar findings were applicable to a diverse group of stimuli that can induce noncanonical autophagy in a FIP200-independent manner. AMDE-1-induced LC3 lipidation was colocalized with the Golgi complex, and was inhibited by the disturbance of Golgi complex. The integrity of the Golgi complex was also required for multiple other agents to stimulate noncanonical LC3 lipidation. These results suggest that the Golgi complex may serve as a membrane platform for noncanonical autophagy where V-ATPase is a key player. V-ATPase inhibitors could be useful tools for studying noncanonical autophagy.

Macroautophagy (hereafter referred to as ‘autophagy') is an evolutionarily conserved process that degrades damaged organelles or long-lived proteins and relies on lysosome system to provide nutrients under conditions of metabolic stress. Disturbance of autophagy has been identified in various human disease such as neurodegenerative disease, infectious disease and cancer.^1, 2^

Autophagy could be mediated by a number of proteins encoded by autophagy-related genes (Atgs). Key ATG molecules are involved in four distinct steps of autophagosome formation and maturation, that is, nucleation, elongation, maturation and fusion.^3^ The nucleation is often initiated by the ULK1 (unc-51-like kinase 1) complex, which includes ULK1, FIP200 and ATG13, and by the autophagy-specific Beclin1 complex, which includes Beclin1, ATG14 and the class III phosphatidylinositol-3 kinase (PI3KC3). These complexes are activated or inhibited by upstream signals, such as those mediated by the mTORC1 and the AMPK pathways.^4^ The initial autophagosomal membranes can be derived from a variety of different sources, which nucleates to form the phagophore with the activation of the above two complexes.^5^ The elongation and maturation of autophagosomal membranes relies on the conjugation systems involving ATG7, ATG12, ATG5, ATG16, ATG10 and ATG3, and eventually yields phosphatidylethanolamine-conjugated LC3 and other ATG8 family members. Finally, the matured autophagosome will fuse with the lysosome for cargo degradation. These steps are known to be essential for the canonical autophagy.

The noncanonical autophagy (NCA) is generally referred to the process that can bypass the involvement of ULK1 and/or Beclin1 complex.^6, 7, 8^ Although an ATG7/ATG5-independent pathway has been reported.^9, 10^ NCA is in general classified as a process dependent on the conjugation system and is characterized by LC3 lipidation. NCA has been identified in various settings. NCA was first found to be independent of the Beclin1 complex.^6, 8, 11^ More recently, proapoptotic compounds such as MK801, gossypol and proteasome inhibitors MG132 and bortezomib have been found to induce Beclin1-independent autophagy.^12^ Autophagy response independent of the ULK1 complex has been reported in response to ammonia,^13^ *cis*-unsaturated fatty acids^14^ and glucose deprivation.^13, 15^

NCA may represent a variety of different LC3-positive structures, including double-membrane autophagosome (by EM), autophagosome-like structure (by GFP-LC3 dots only), noncanonical LC3 lipidation (by western blot assay only) and LAP (LC3-associated phagocytosis, by GFP-LC3 dots).^16, 17^ LAP may be a special form of NCA but has connections with host autophagic responses in terms of microbial infection.^16^ NCA structures with either single or double membranes have the same function as the canonical autophagy in sequestering cytoplasm and compartmentalizing invading pathogens, which are ultimately degraded in the lysosomal compartment.^7^

The function of NCA is not very clear in every case. However, some processes such as entosis and LAP have been well studied,^18, 19, 20^ suggesting that the noncanonical functions of ATG molecules have important physiological functions. Phagocytosis of live or dead cells or pathogens can be facilitated by the LC3-positive compartment.^20, 21^ In addition, chemical-induced NCA or noncanonical LC3 lipidation has been reported to be positively correlated with human tumor cell death^8, 22^ and resistance to pathogens.^23^ It is possible that cells lacking ULK1 or Beclin1 activity, or with a low autophagy-initiating capability might evolve to use NCA as a way to resist pathogen invasion or to survive in adverse environments. NCA might be either an important complementary mechanism or a parallel system for the regulation of a list of pathophysiological processes.

How NCA is activated is not well understood in many cases, which hampers the understanding of the function of NCA and its relationship with canonical autophagy. AMDE-1 is a novel chemical that can trigger canonical autophagy.^24^ In this study, we demonstrated that AMDE-1 can also induce NCA, which depends on vacuolar-type H^+^-ATPase (V-ATPase) and an integrated Golgi complex. Furthermore, we showed that these requirements were generally needed for NCA induced by several other agents, thus revealing a potentially general mechanism for a diverse set of NCA stimuli.

## Results

### AMDE-1-triggered autophagy is independent of the ULK1 complex but dependent on ubiquitin-like conjugation systems

**Figure 1:**
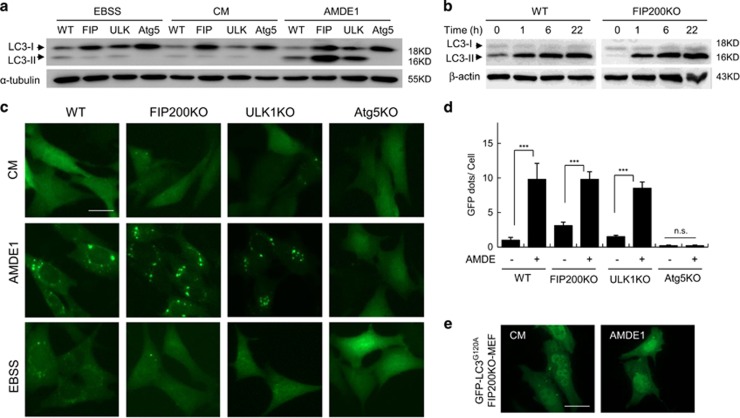
AMDE-1 can induce NCA, which is independent of FIP200 and ULK1 but dependent on the conjugation system. (**a**) Wild- type MEFs (WT), FIP200KO-MEFs (FIP), ULK1KO-MEFs (ULK) and Atg5KO-MEFs (Atg5) were treated with AMDE-1 (10 *μ*M) for 6 h, or EBSS for 2 h, and then analyzed by immunoblotting. (**b**) Immunoblotting analysis of WT-MEFs and FIP200KO-MEFs treated by AMDE-1 (10 *μ*M) at different time points. (**c**) WT-MEFs, FIP200KO-MEFs, ULK1KO-MEFs and Atg5KO-MEFs expressing GFP-LC3 were treated with or without AMDE-1 (10 *μ*M) for 6 h, or EBSS for 2 h and then assessed for LC3 puncta formation (green). (**d**) Qualification of GFP-LC3 puncta in (**c**). (**e**) FIP200KO-MEFs expressing GFP-LC3^G120A^ were treated with or without AMDE-1 (10 *μ*M) for 6 h and then assessed for LC3 puncta formation (green). ***P<0.001; NS, not significant. Bar=25 *μ*m

AMDE-1 was found through high-content screening using GFP-LC3.^24^ It is a novel autophagic modulator that can initiate canonical autophagy at the early stage via suppression of mTORC1. We used MEFs deficient in FIP200 or ULK1 to investigate the role of the ULK1 complex in AMDE-1-induced autophagy. Surprisingly, AMDE-1 still induced LC3-II formation in FIP200KO-MEF and in ULK1KO-MEF (Figure 1a). The kinetics of AMDE-1-induced LC3-II formation in FIP200KO-MEF was the same as that in WT-MEF (Figure 1b). The number of GFP-LC3 puncta in FIP200KO-MEF and in ULK1KO-MEF was reduced but remained significant following AMDE-1 stimulation (Figures 1c and d). When GFP-LC3^G120A^ was applied, which is defective for LC3 lipidation, no obvious GFP puncta were elicited after treatment (Figure 1e), suggesting that AMDE-1-induced GFP puncta in FIP200KO-MEF involved the lipidation of GFP-LC3.

Nevertheless, AMDE-1-induced LC3 lipidation required the canonical conjugation systems. Thus, there was no LC3-II formation (Figure 1a) nor GFP-LC3 puncta (Figure 1c and d) in Atg5KO-MEF following the treatment with AMDE-1. Consistently, Atg7, Atg4B and Atg3 were all required for AMDE-1-induced LC3 lipidation (Supplementary Figure S1). Taken together, these data indicated that AMDE-1 was able to trigger NCA in FIP200KO and ULK1KO cells, which was dependent on the ubiquitin-like conjugation systems.

### AMDE-1-induced autophagy does not require Beclin1 complex

**Figure 2:**
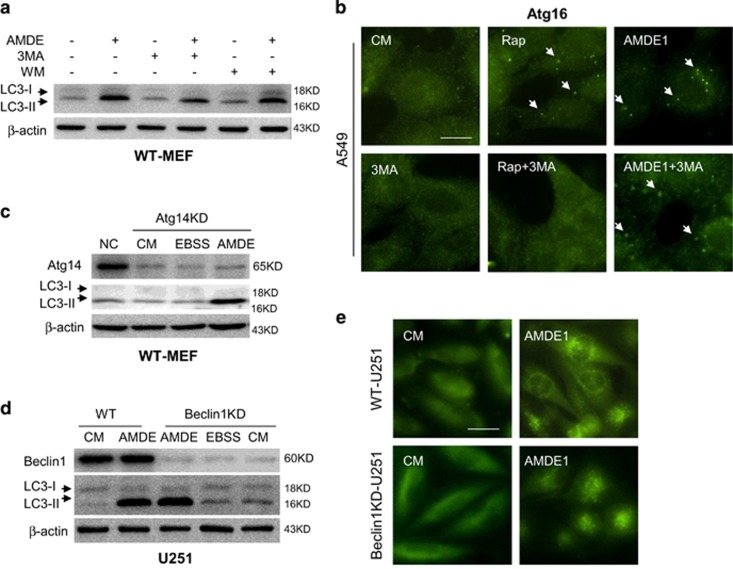
AMDE-1-induced NCA does not require the participation of the canonical autophagy-specific class III PI-3 kinase. (**a**) WT-MEFs were treated by AMDE-1 (10 *μ*M) with or without 3-MA (10 mM) or WM (1 *μ*M) for 6 h, and then analyzed by immunoblotting. (**b**) A549 cells were treated as indicated (Rap, 1 *μ*M; AMDE-1, 10 *μ*M; 3-MA, 10 mM) for 6 h, followed by immunostaining with an anti-ATG16L1 antibody. Arrows indicate ATG16-positive dots. (**c**) Atg14-siRNA was transfected to wild- type MEFs for 48 h, followed by treatment with AMDE-1 or EBSS. (**d** and **e**) Wild-type and Beclin1 KD U251 cells expressing GFP-LC3 were treated with or without AMDE-1 (10 *μ*M) for 6 h or EBSS for 2 h and then assessed for LC3-II formation by immunoblotting (**d**), or immunostaining (**e**). Bar=25 *μ*m

To evaluate whether the observed LC3 lipidation was initiated via Beclin1 complex, we used PI3KC3 inhibitors 3-methyladenine (3-MA) and wortmannin (WM) and found that LC3 lipidation could not be suppressed by these canonical autophagy inhibitors (Figure 2a). We further checked the formation of Atg16 puncta, a critical event that occurs earlier than LC3 lipidation. As shown in Figure 2b, rapamycin induced strong Atg16 punctuation, which was totally suppressed by a PI3KC3 inhibitor 3-MA, whereas AMDE-1-induced Atg16 puncta failed to be suppressed by 3-MA. Atg14 is an important component that bridges Beclin1 and Vps34 to allow formation of Beclin1 complex.^25^ Similar to the pattern of PI3KC3 inhibition, knockdown (KD) of Atg14 could not impair the formation of LC3-II induced by AMDE-1 (Figure 2c). Finally, we found that there was no difference in LC3-II conversion and GFP-LC3 puncta formation between wild-type U251 cells and U251 cells with constitutive Beclin1 KD (Figure 2d and e). These data suggested that Beclin1 complex was not required for AMDE-1-induced NCA.

### AMDE-1 does not recruit WIPI2 but does recruit Atg16 and Atg12

**Figure 3:**
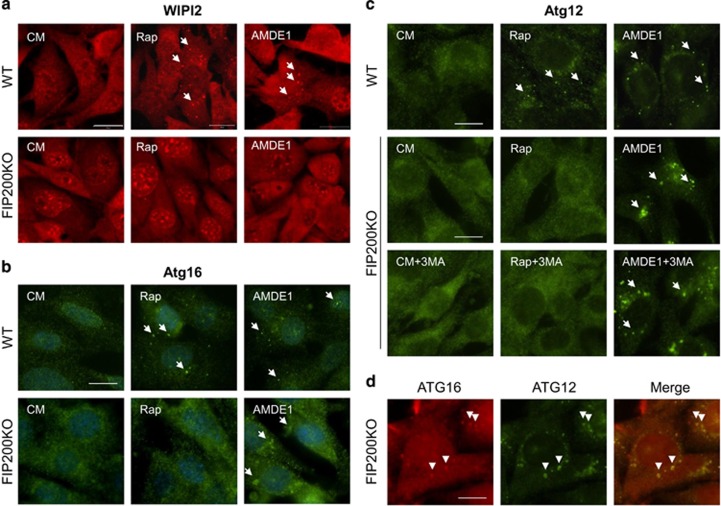
AMDE-1-induced NCA does not recruit WIPI2, but Atg16 and Atg12. (**a**) WT-MEFs and FIP200KO-MEFs were treated with or without AMDE-1 (10 *μ*M) or rapamycin (Rap, 1 *μ*M) for 6 h and then assessed for WIPI2 staining in the cytosol (Red). (**b** and **c**) WT-MEFs and FIP200KO-MEFs were treated as indicated (Rap, 1 *μ*M; AMDE-1, 10 *μ*M; 3-MA, 10 mM) for 6 h, followed by immunostaining of ATG16 (**b**) and ATG12 (**c**). Arrows indicate the examples of WIPI2-, ATG12- and ATG16-positive dots. (**d**) Costaining of ATG12 (green) and ATG16 (red) in FIP200KO-MEF after AMDE-1 treatment for 6 h. Arrowheads indicate the examples of colocalized dots of ATG12 and ATG16. Bar=25 *μ*m

WIPI2, a phosphoinositide-interacting protein with WD repeat domain, and DFCP1, a double FYVE-containing protein 1, were monitored to confirm the initiation of autophagy. WIPI2 forms a complex with ATG2 and functions in autophagosome formation in a phosphatidylinositol-3-phosphate (PI3P)-dependent manner, perhaps by recruiting ATG16 for LC3 lipidation.^26^ In the absence of FIP200, there was no WIPI2 puncta formed following either rapamycin or AMDE-1 treatment (Figure 3a), and there was no DFCP1-positive structures as well (Supplementary Figure S2), suggesting that WIPI2 and DFCP1 were strictly regulated by ULK1 complex and was recruited by AMDE-1. The data also suggested that WIPI2 and DFCP1 could be dispensable in AMDE-1-induced NCA.

On the contrary, both rapamycin and AMDE-1 induced ATG16 puncta in wild-type MEFs, but such puncta could be induced only by AMDE-1 in FIP200KO-MEFs (Figure 3b). Staining for ATG12-positive structures showed that the staining pattern was similar to that of ATG16 (Figure 3c). ATG12-positive dots in FIP200KO-MEF was not suppressed by 3-MA. We then compared the distribution of ATG12- and ATG16-positive dots, and the conjugation of ATG5–ATG12 after AMDE-1 treatment. Nearly 100% of ATG12 dots were colocalized with ATG16 dots (Figure 3d). ATG12–ATG5 conjugation was not affected (Supplementary Figure S3), suggesting that the integrity of ATG5–12/16 complex remained in AMDE-1 treatment. These data suggested that AMDE-1 could recruit the ATG5–12/16 complex to a membrane platform for NCA via a mechanism independent of WIPI2 and/or DFCP1.

### Bafilomycin blocks AMDE-1-induced NCA

**Figure 4:**
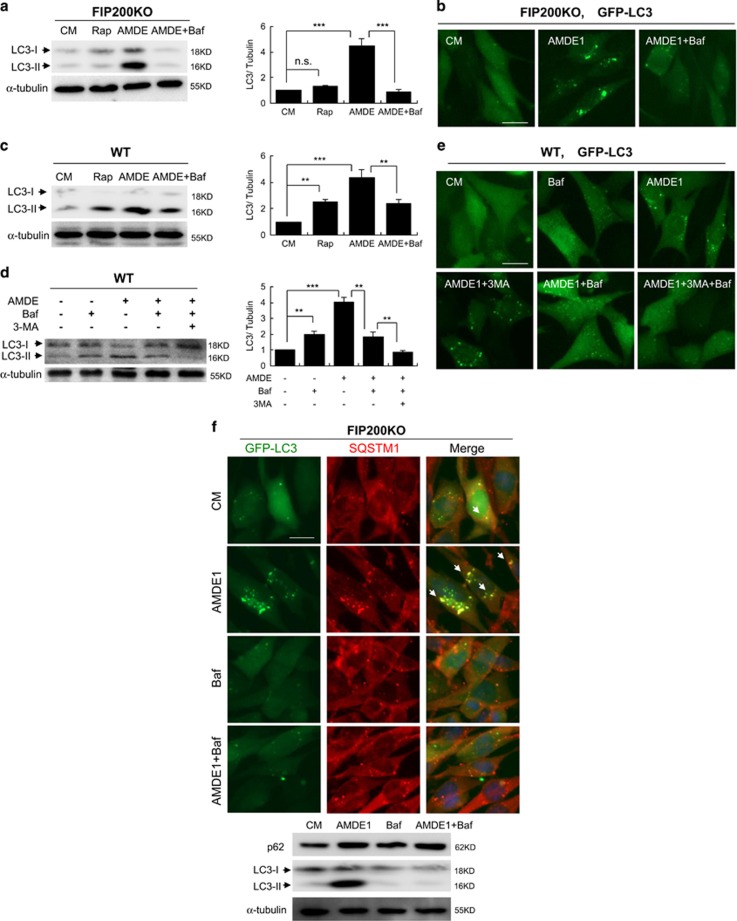
AMDE-1-induced NCA can be suppressed by Baf. (**a**) FIP200KO MEFs were treated with or without AMDE-1 (10 *μ*M), Rap (1 *μ*M) and/or Baf (0.5 *μ*M) for 6 h, and then analyzed by immunoblotting. (**b**) FIP200KO MEFs expressing GFP-LC3 were treated as indicated (AMDE-1, 10 *μ*M; Baf, 0.5 *μ*M) for 6 h, and then assessed for LC3 puncta formation. (**c** and **d**) Wild-type MEFs were treated as indicated (AMDE-1, 10 *μ*M; Rap, 1 *μ*M; 3-MA, 10 mM; Baf, 0.5 *μ*M) for 6 h, and then analyzed by immunoblotting. The LC3-II/Tubulin ratio was calculated relative to that of control medium (CM). (**e**) Wild-type MEFs expressing GFP-LC3 were treated as indicated (AMDE-1, 10 *μ*M; 3-MA, 10 mM; Baf, 0.5 *μ*M) for 6 h, and then assessed for LC3 puncta formation. (**f**) FIP200KO-MEFs expressing GFP-LC3 were treated by AMDE-1 (10 *μ*M) with or without Baf (0.5 *μ*M) for 6 h, followed by immunostaining of SQSTM1. Arrows indicate the examples of colocalized puncta of GFP-LC3 and SQSTM1. Bar=25 *μ*m. **P<0.01; ***P<0.001

We found that a commonly used autophagy degradation inhibitor, bafilomycin A_1_ (Baf), was able to inhibit AMDE-1 induced LC3 lipidation in FIP200KO-MEFs (Figures 4a and b). In wild-type MEFs, Baf alone was able to reduce AMDE-1 induced LC3-II formation (Figure 4c). Notably, the combination of 3-MA and Baf almost completely repressed LC3-II formation (Figure 4d) and GFP-LC3 puncta (Figure 4e) in wild-type MEF. These data indicated that AMDE-1 induced autophagy in wild-type MEF could be caused by two different mechanisms, one was for canonical autophagy, which was inhibited by 3-MA, and the other was for NCA, which was suppressed by Baf.

SQSTM1/p62 is a multifunctional adaptor protein that recruits ubiquitinated protein substrates to LC3 for autophagic degradation.^27^ The puncta of SQSTM1 increased markedly in response to AMDE-1 in FIP200KO-MEF, in which SQSTM1 overlapped with LC3 nearly completely (Figure 4f). In line with the fate of LC3 puncta, the accumulated SQSTM1 puncta were abolished by Baf as well (Figure 4f). Such AMDE-1-induced colocalization also occurred in HeLa cells even in the presence of 3-MA (Supplementary Figure S4). To investigate whether SQSTM1 was involved in LC3 lipidation, we knocked it down and found reduction of SQSTM1 could not decrease the overall number of LC3 puncta, and the LC3-II level induced by AMDE-1, regardless of the presence of 3-MA (Supplementary Figure S4). These data thus indicated that SQSTM1 was not required for AMDE-1-induced LC3 lipidation.

### V-ATPase inhibitors instead of lysosome inhibitors and ionophores block LC3 lipidation

**Figure 5:**
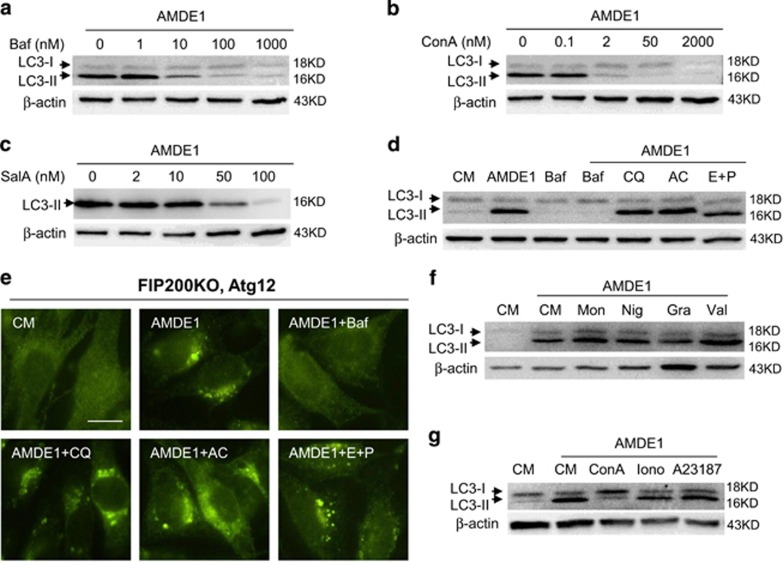
AMDE-1-induced NCA involves V-ATPase, but not lysosome activity or intracellular ion redistribution. (**a**–**c**) FIP200KO-MEFs were treated with AMDE-1 (10 *μ*M) in the presence or absence of Baf, ConA or SalA for 6 h, followed by immunoblotting analysis for LC3. (**d**–**g**) FIP200KO-MEFs were treated as indicated for 6 h, followed by immunoblotting analysis (**d**, **f** and **g**) and ATG12 immunostaining (**e**). AMDE-1 (10 *μ*M), Baf (0.5 *μ*M), AC (20 mM), CQ (40 *μ*M), E64d (25 *μ*M) plus pepstatin A (50 *μ*M), monesin (Mon, 10 *μ*M), nigarecin (Nig, 1.5 *μ*M), gramicidin A (Gra, 5 *μ*M), valinomycin (Val, 10 *μ*M), ConA (0.2 *μ*M), ionomycin (Iono, 2 *μ*M) and A23187 (2 *μ*M). Bar=25 *μ*m

Similar to Baf, concanamycin A (ConA) is a well-studied V-ATPase inhibitor. Salicylihalamide A (SalA) is another mammalian-specific V-ATPase inhibitor, but has a distinct binding pattern compared with Baf and ConA.^28^ Similar to Baf, both ConA and SalA suppressed AMDE-1-induced LC3-II significantly in FIP200KO-MEF at the dose from 1 to 100 nM (Figures 5a–c), which indicated that V-ATPase was a critical molecule for NCA and the inhibitory effect of NCA by Baf was indeed targeted toward this molecule.

V-ATPase is significantly present in the lysosomal membrane and is responsible for the acidic environment of the lysosome. In fact, Baf is often used to disrupt autophagic and non-autophagic lysosomal degradation through its inhibition on lysosomal V-ATPase.^29^ To determine whether the lysosome acidic or proteolytic environment was required for AMDE-1-induced NCA, we assessed other well-known lysosome inhibitors, including chloroquine (CQ), ammonia chloride, and protease inhibitors E64d and pepstatin A. We found that none of these chemicals could suppress AMDE-1-induced NCA based on the results of immunoblotting for LC3-II (Figure 5d) and immunostaining for ATG12 puncta (Figure 5e). These observations suggested that lysosome V-ATPase could be affected by Baf, ConA or SalA, but lysosomal functional change might not be the cause leading to NCA in AMDE-1 treatment.

In addition, Baf has been reported to have the activity as a K^+^ ionophore.^30^ To determine whether redistribution of intracellular ions has a general impact on AMDE-1-induced NCA, we assessed the effects of several ionophores, including monensin (for Na^+^), nigericin (for K^+^), valinomycin (for K^+^), gramicidin A (for monovalent cations), ionomycin (for Ca^2+^) and A23187 (for Ca^2+^). The results indicated that these chemicals did not block AMDE-1-induced LC3 lipidation (Figures 5f–g).

### Functional V-ATPase is required for noncanonical LC3 lipidation

**Figure 6:**
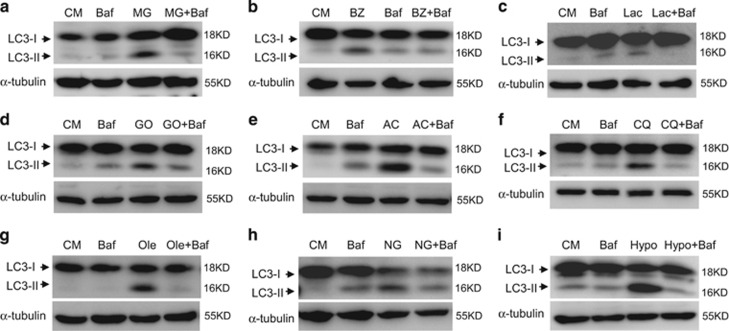
Baf can inhibit NCA induced by multiple agents. Immunoblotting analysis of FIP200KO-MEFs treated with MG132 (5 *μ*M) for 12 h (MG, **a**), bortezomib (30 nM) for 12 h (BZ, **b**), lactacystin (5 *μ*M) for 12 h (Lac, **c**), gossypol (20 *μ*M) for 12 h (GO, **d**), ammonia chloride (10 mM) for 24 h (AC, **e**), CQ (100 *μ*M) for 6 h (CQ, **f**), sodium oleate (500 *μ*M) for 6 h (Ole, **g**), glucose deprivation for 24 h (NG, **h**) or hypotonic buffer for 1 h (Hypo, **i**) with or without Baf (0.5 *μ*M)

NCA could be induced by a variety of chemicals.^12^ Recently, chemicals such as proteasome inhibitors (MG132, bortezomib and lactacystin), ammonia, gossypol and CQ were reported to promote the lipidation of LC3 in a ULK1- independent or Beclin1-independent manner.^12, 31^ Whether these chemicals had any characteristics in common in regulating noncanonical LC3 lipidation processes was unknown. We found Baf impaired LC3 lipidation caused by these chemicals (Figures 6a–f). Furthermore, unsaturated fatty acids, glucose deprivation and osmotic imbalance were also reported to induce NCA.^13, 14, 31^ We found that Baf inhibited LC3 lipidation in FIP200KO cells cultured with solium oleate, in low glucose medium or in hypotonic medium (Figures 6g–i). These data suggested that V-ATPase could mediate a mechanism for NCA common to many different stimuli.

### V-ATPase-positive Golgi complex is involved in LC3 lipidation

**Figure 7:**
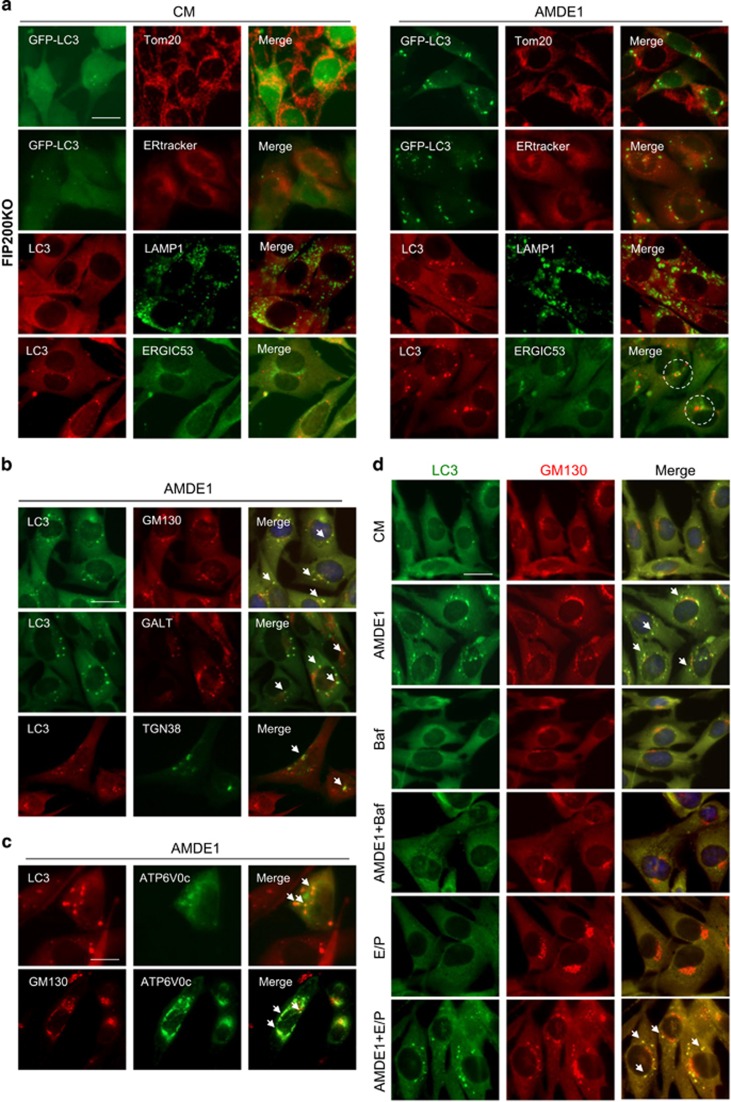

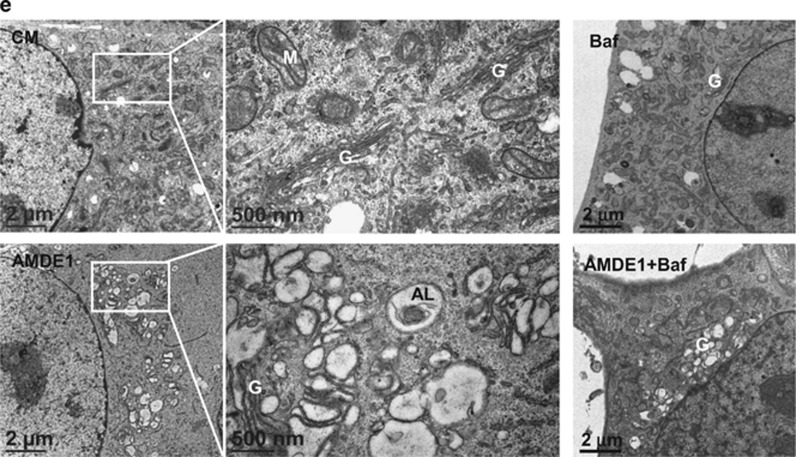
AMDE-1-induced NCA involves the Golgi complex. (**a**) FIP200KO-MEFs with or without expressing GFP-LC3 were treated with AMDE-1 (10 *μ*M) followed by immunostaining for LC3, Tom20, ERtracker, LAMP1 or ERGIC-53. Dotted circles indicate the juxtaposed LC3 and ERGIC-53 signals. (**b** and **c**) FIP200KO-MEFs with or without expressing mCherry-GALT, GFP-ATP6V0c and CFP-TGN38 were treated with AMDE-1 (10 *μ*M) followed by immunostaining for LC3, or GM130. Arrows indicate colocalization of LC3 with GM130, GALT and TGN38 (**b**), or ATP6V0c with LC3 or GM130 (**c**). (**d** and **e**) FIP200KO-MEFs were treated by AMDE-1 (10 *μ*M) with or without Baf (0.5 *μ*M), or E64D (25 *μ*M) plus pepstatin A (50 *μ*M) for 6 h, followed by immunostaining for LC3 and GM130. Arrows indicate colocalization of LC3 with GM130 (**d**). Cells were also analyzed by transmission electron microscopy (**e**). AL, autolysosome; G, Golgi complex; M, mitochondria. Bar=25 *μ*m

As Baf and other V-ATPase inhibitors did not seem to act upon the lysosome for its inhibition of NCA, their effect on NCA might involve other intracellular membrane compartments where V-ATPase is present. To determine potential contributions of these compartments to AMDE-1-induced NCA, we first assessed whether AMDE-1-induced LC3 puncta would be colocalized with these subcellular organelles in FIP200KO-MEFs. As shown in Figure 7a, AMDE-1-induced LC3-positive structures did not overlap with the mitochondria marker Tom20, the lysosome marker LAMP1 and the ER marker ERtracker, but were juxtaposed to the ER to Golgi intermediate compartment marker, ERGIC-53. Interestingly, most of the LC3 puncta were significantly overlapped with the *cis*-Golgi matrix marker GM130 and the *trans*-Golgi membrane marker *β*-1, 4-galactosyltransferase (GALT), and partially colocalized with *trans*-Golgi network marker, TGN38 (Figure 7b), suggesting that Golgi complex might be the main membrane platform for AMDE-1-induced LC3 lipidation. Transient overexpression of V0 complex subunit c of V-ATPase, ATP6V0c, the specific target of Baf and ConA, promoted the colocalization of V-ATPase with LC3, and of V-ATPase with GM130 after AMDE-1 treatments (Figure 7c). Consistently, lysosomal inhibitors, E64D/pepstatin A (Figure 7d), or CQ (Supplementary Figure S5), did not seem to affect the distribution of AMDE-1-induced LC3 puncta in the Golgi complex. E64D/pepstatin A did not cause GFP-LC3 puncta nor did the combination suppressed AMDE-1-induced LC3 puncta (Figure 7d).

Compared with the control cells where the Golgi complex was mostly a focal unit adjacent to the nucleus, AMDE-1 treatment seemed to disperse the Golgi complex (Figures 7b–d). Electron microscopic analysis indicated that there were increased classical autophagosomes and autolysosome structures (Supplementary Figure S6), and notably swollen Golgi complex delimited by single-membrane in AMDE-1-treated cells (Figure 7e). Furthermore, Baf did not seem to be able to change the dispersed pattern of the Golgi complex (Figure 7d) nor the swelling of the Golgi complex (Figure 7e) in AMDE-1-treated cells. Thus, the V-ATPase was not likely involved in the structural alteration of the Golgi complex, but was likely involved in a separate step of recruitment of LC3 to the Golgi membranes.

### An integrated Golgi complex is required for AMDE-1-induced LC3 lipidation

**Figure 8:**
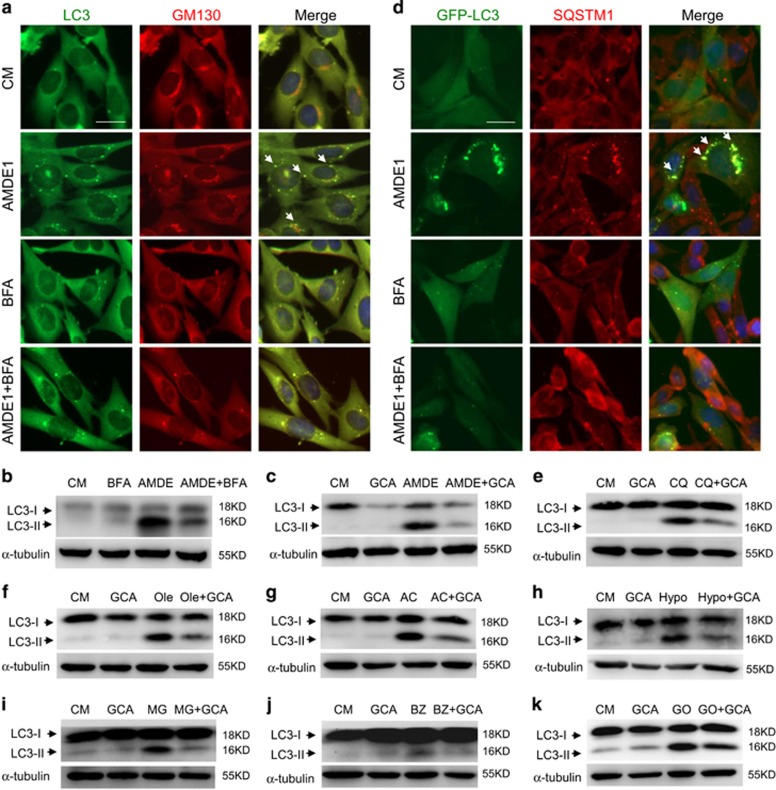
AMDE-1-induced NCA can be blocked by Golgi toxins. (**a**) FIP200KO-MEFs were treated by AMDE-1 (10 *μ*M) with or without BFA (5 *μ*g/ml) for 6 h, followed by immunostaining for LC3 and GM130. Arrows indicate colocalization of LC3 with GM130. (**b**). FIP200KO-MEFs expressing GFP-LC3 were treated by AMDE-1 (10 *μ*M) with or without BFA (5 *μ*g/ml) for 6 h, followed by immunostaining of SQSTM1. Arrows indicate colocalization of GFP-LC3 with SQSTM1. (**c** and **d**) FIP200KO-MEFs were treated by AMDE-1 (10 *μ*M) with or without BFA (5 *μ*g/ml) or GCA (20 *μ*M) for 6 h, followed by immunoblotting analysis. (**e**–**k**) Immunoblotting analysis of FIP200KO-MEFs treated with CQ (100 *μ*M, 6 h, **e**), Ole (500 *μ*M, 6 h, **f**), AC (10 mM, 24 h, **g**), hypotonic buffer (Hypo, 1 h, **h**), BZ (30 nM, 12 h, **i**), MG132 (MG, 5 *μ*M, 12 h, **j**), and GO (20 *μ*M, 12 h, **k**) with or without GCA (20 *μ*M). Bar=25 *μ*m

It is possible that an integrated Golgi complex may actually be required for AMDE-1 to induce noncanonical LC3 lipidation. To test this hypothesis, we used two well-established Golgi toxins, brefeldin A (BFA) and golgicide A (GCA). Both are known to inhibit ER to Golgi vesicle trafficking and to fragment the Golgi complex.^32^ We found that BFA and GCA not only weakened the staining signal of Golgi complex (Figure 8a) but also abolished AMDE-1-induced LC3 puncta and LC3-II formation (Figures 8a–c). Consistently, SQSTM1, which were recruited by LC3 following AMDE-1 treatment (Figure 4 and Supplementary Figure S4), failed to accumulate in the presence of BFA (Figure 8d), suggesting that the Golgi complex was an important platform for AMDE-1-induced LC3 lipidation and SQSTM1 recruitment.

We then asked whether the Golgi toxin could suppress NCA caused by other stimuli. Indeed, GCA suppressed LC3-II formation in FIP200KO cells induced by CQ, sodium oleate, AC, hypotonic buffer and three proteasome inhibitors (MG132, bortezomib and gossypol) (Figures 8e–k). Taken together, these observations indicated that an integrated Golgi complex was required for many NCA stimuli to promote noncanonical LC3 lipidation.

## Discussion

Here we demonstrated that AMDE-1 was able to induce NCA, in the absence of ULK1 complex or Beclin1/Atg14 complex. A form of Atg7- or Atg5-independent NCA had been reported in which LC3 lipidation did not occur.^9^ However, most types of NCA including LAP still require the conjugation system and can be detected by LC3 lipidation assay. AMDE-1-induced NCA requires the LC3 conjugation system. SQSTM1 is an adaptor molecule that links autophagosomal membranes to the ubiquitinated targets to facilitate target degradation. In our study, accumulated LC3 was overlapped with SQSTM1, but did not depend on the latter. The colocalization of the two molecules suggests that SQSTM1 might still act as an adaptor molecule in AMDE-1-induced NCA for target clearance.

In canonical autophagy, the ULK1 complex or the Beclin1/Atg14 complex is required to couple the canonical autophagy stimuli, such as amino-acid deprivation, to the generation of PI3P on specific membranes where LC3 lipidation can subsequently occur. Activation of NCA could be related to the bypass of ULK1 complex for Beclin1/Atg14 activation, or be related to the independence of LC3 lipidation on PI3P. Recently, PI5P was found to regulate autophagy and sustain NCA in PI3P-depleted cells.^33^ The importance of PI3P or PI5P generation in autophagy may depend on the type of stimuli or specific membranes where LC3 lipidation occurs. AMDE-1-induced NCA can occur in the absence of ULK1 complex or the Beclin1/Atg14 complex, suggesting that PI3P generation may not be required. AMDE-1 might use alternative mechanisms to activate the two ubiquitin-like conjugation systems to initiate the NCA. This hypothesis is also implicated in the different morphology of the ATG12 and ATG16 puncta. When rapamycin was applied to wild-type cells, ATG12 dots were small but bright. In contrast, AMDE-1-induced ATG12 dots looked larger but less intensive in FIP200KO cells (Figure 3).

As NCA represent distinct processes, the membrane sources or the biogenesis of LC3-positive structures could be different from those of canonical autophagy. In canonical autophagy, the ER membrane, where the DFCP1-positive omegasome is derived to give rise to the autophagosomes, seems to be the major membrane source. However, the Golgi complex, mitochondria–ER contact sites and plasma membrane-derived endocytic organelles can also contribute to autophagosomes biogenesis.^34^ The source of membranes on which LC3 lipidation occurs could be also diverse in NCA, which can give rise to either double- or single-membrane vesicles.^7^

**Figure 9:**
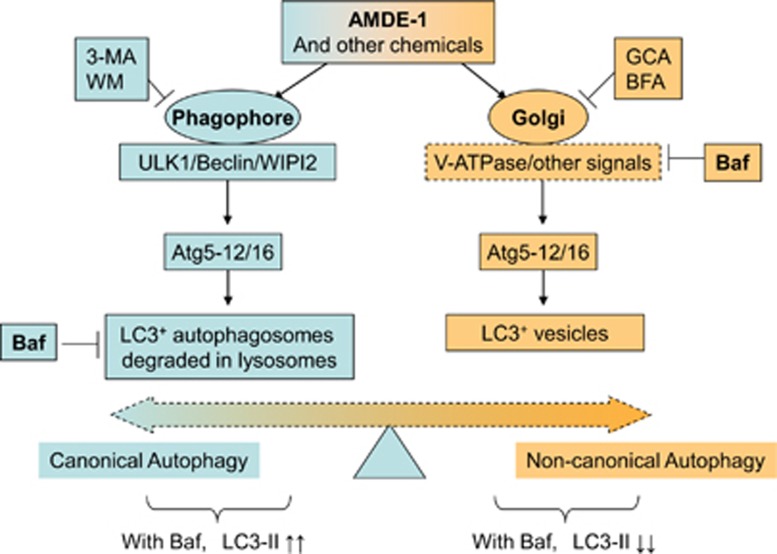
The working model of AMDE-1-induced canonical and noncanonical LC3 lipidation. AMDE-1 is able to induce both canonical autophagy and NCA. The ULK1 complex and the Beclin1–Atg14 complex are required in the canonical pathway, but not in the noncanonical pathway. However, the LC3 conjugation system is required in both pathways. While the phagophore provides the membrane platform for LC3 lipidation in canonical autophagy, the Golgi complex is likely the membrane platform for the noncanonical LC3 lipidation. Notably, the V-ATPase can have an important role in the latter by acting at the Golgi complex, although the exact molecular mechanism has yet to be determined. Thus, V-ATPase inhibitors, such as Baf, can cause LC3-II accumulation because of blockage of autolysosome degradation in canonical autophagy, but can cause LC3-II decrease because of suppression of Golgi signaling at the early stage in NCA. Different agents can thus affect the canonical and noncanonical pathways, which modulate the ultimate outcomes for cells treated with chemicals with dual capability, such as AMDE-1

Among the various membrane structures, the Golgi complex deserves a particular attention as it could serve as a common platform for canonical autophagy and NCA.^35^ The disruption of Golgi complex by genetic intervention of *α*SNAP triggers Beclin1-independent autophagy.^36^ An intact Golgi complex is also found to be required for unsaturated fatty acid-induced NCA.^14^ The structure collapse of the Golgi complex induced by BFA inhibits *cis*-unsaturated fatty acid-triggered autophagy. BFA and GCA also abolish AMDE-1-induced NCA. In addition, AMDE-1-induced LC3 puncta are colocalized with the Golgi complex, but not with other subcellular organelles. AMDE-1 was able to cause swelling of Golgi complex. All these data support that AMDE-1 could target the Golgi complex where LC3 lipidation is triggered (Figure 9). Although BFA was previously found to induce canonical autophagy in wild-type cells through ER stress,^37^ we did not observe any LC3 lipidation in FIP200KO cells treated with BFA or GCA (data not shown). Thus, these agents cause different effects in canonical autophagy and NCA.

Lysosomes can be an important organelle promoting NCA and where noncanonical LC3 lipidation occurs.^13, 14, 31^ However, a diverse group of lysosome inhibitors with different mechanisms did not affect AMDE-1-induced noncanonical LC3-II formation (Figure 5d), GFP-LC3 punctation (Figure 7d and Supplementary Figure S5) and Atg12 puncta formation (Figure 5e). Although CQ alone could induce NCA^13, 14, 31^ (Figures 6f and Supplementary Figure S5), this might not suggest that the disruption of lysosome function was sufficient to cause NCA, as E64D/pepstatin A could not induce NCA (Figure 7d). Notably, CQ (Supplementary Figure S5) or E64D/pepstatin A (Figure 7d) could not inhibit AMDE-1-induced LC3 puncta and their colocalization with the Golgi complex. In contrast, CQ-induced noncanonical LC3 puncta seemed to be colocalized with the Golgi marker (Supplementary Figure S5). Taken together, while the involvement of the lysosome and other membranes in AMDE-1-induced NCA cannot be excluded, our current data at least suggest that simply inhibiting lysosome function may not be sufficient to inhibit AMDE-1-induced NCA, whereas the Golgi complex could be a common platform for noncanonical LC3 lipidation under a variety of conditions.

The involvement of V-ATPase in NCA has been reported in a previous study.^13, 14, 31^ Here we report that three different inhibitors of V-ATPase, Baf, ConA and SalA, suppresses NCA induced by AMDE-1 and several other agents, which had not been known to depend on V-ATPase for their NCA activity. We have further found that although V-ATPase is located at different endosomal compartments,^38^ the inhibition is not likely related to the dysfunction of the lysosome or redistribution of intracellular ions because chemicals directly disturbing these functions have no effects on AMDE-1-induced NCA (Figure 5,Figure 7d and Supplementary Figure S5). Our studies instead indicate that the suppressive effect of Baf is most likely related to Golgi-located V-ATPase, considering the involvement of the Golgi complex in the early stage of AMDE-1-induced NCA. Thus, AMDE-1 may act on the Golgi complex, which then engages V-ATPase to activate the LC3 conjugation system, leading to LC3 lipidation on the Golgi membranes (Figure 9).

There are few known inhibitors for NCA. Identification of V-ATPase inhibitors as NCA inhibitors for a group of diverse stimuli could facilitate the understanding of the mechanism. It has to be pointed out that V-ATPase inhibitors, such Baf, can be inhibitory to both canonical autophagy at the late stage and NCA at the early stage. However, the effect on the detection of LC3-II and LC3 puncta is different. While reducing LC3-II and LC3 puncta in NCA, these inhibitors, such as the traditional lysosome inhibitors, cause accumulation of LC3-II and LC3 puncta as the results of the blockage of lysosome degradation.^1, 39^ Thus, V-ATPase inhibitors might be important for dissecting signaling mechanisms through or bypass key Atgs for stimuli, such as AMDE-1, which can cause both canonical autophagy and NCA in the same cells. For these stimuli, it will require the inhibition of both pathways to fully suppress LC3 lipidation (Figures 4 and 9).

In summary, we have defined a new effect of AMDE-1 in NCA in the absence of ULK1 complex or Beclin1 complex. The NCA triggered by AMDE-1 requires functional V-ATPase and an integrated Golgi complex. This seems to be a common mechanism for a number of diverse NCA stimuli. Our finding may provide important information on the membrane source for, and the initiation mechanism of, the NCA.

## Materials and Methods

### Chemicals and antibodies

AMDE-1 was purchased from Key Organics (Bedford, MA, USA). A23187, ammonia chloride, BFA, CQ, ConA, 3-MA, gramicidin, monensin, valinomycin, nigericin and HgCl_2_ were from Sigma-Aldrich (St. Louis, MO, USA). Baf, bortezomib and WM were from LC Laboratories (Woburn, MA, USA). Rapamycin, E64D and pepstatin A were from AG Scientific (San Diego, CA, USA). MG132, GCA were from Selleckchem (Houston, TX, USA). Sodium oleate was from Sangon (Shanghai, China). Lactacystin was from Cayman (Ann Arbor, MI, USA). Gossypol was from Meilunbio (Dalian, China). Lipofectamine 2000 was from Invitrogen (Carlsbad, CA, USA). SalA was a gift from Dr De Brabander (UT Southwestern, Dallas, TX, USA). The siRNA for human SQSTM1/p62 (5′-GCATTGAAGTTGATATCGATTT-3′) and the scrambled control (5′-UUCUCCGAACGUGUCACGU-3′) were obtained from Ruibo (Guangzhou, China).

Antibodies to ERGIC-53 (no. sc-398777), GFP (no. sc-9996) and Tom20 (no. sc-11415) were from Santa Cruz (Santa Cruz, CA, USA); antibodies to *β*-actin (no. 3700), ATG3 (no. 3415), ATG12 (no. 2011S), ATG7 (no. 8558), calnexin (no. 2679) were from Cell Signaling Technology (Danvers, MA, USA); antibodies to SQSTM1 (no. PM045), MAP1LC3B (no. PM036), ATG4B (no. M134) and ATG16L1 (no. M150) were from MBL International (Woburn, MA, USA); antibodies to ATG5 (no. A0731), LC3B (no. L7543), *α*-tubulin (no. T6074) were from Sigma (St. Louis, MO, USA); antibody to WIPI2 (no. ab105459) was from Abcam (Cambridge, UK); antibody to GM130 (no. BD-610822) was from BD Bioscience (San Jose, CA, USA); antibody to TGN46 (no. AHP500GT) was from AbDSerotec (Oxford, UK). Secondary antibodies conjugated with Alexa Fluor-488, Alexa Fluor-594 or horseradish peroxidase (no. 7074) were from Invitrogen and Cell Signaling Technology, respectively.

### Cell culture and transfection

Wild-type and ATG5- ATG3-, ATG7-, ATG4b-, ULK1-, FIP200-deficient MEFs, A549, U251 and Beclin1 KD U251 cells had been described previously.^40, 41^ Cells were cultured at 37 °C in a humidified air atmosphere with 5% (v/v) CO_2_. DMEM with or without glucose (Thermo Scientific, Rockford, IL, USA) was supplemented with 10% (v/v) of fetal bovine serum (Gibico, Grand Island, NY), USA and other standard supplements. EBSS (Thermo Scientific) with no serum and other supplements was used as starvation medium. Hypotonic buffer was prepared by diluting DMEM with water (water/DMEM: 3 : 1).

For transient expression, cells were cultured in 24-well plates and were transfected with DNA constructs using Lipofectamine 2000 (Invitrogen) and analyzed 24–48 h later. For gene KD, siRNA (120 nM) was transfected into 1 × 10^6^ cells using Oligofectamine (Invitrogen) for 48–72 h before analysis.

### Immunoblotting assays

Cell lysates were prepared with RIPA buffer with a protease and phosphatase inhibitor cocktail (Thermo Scientific). Twenty to thirty micrograms of total protein were separated by SDS-PAGE and transferred to PVDF membranes (Millipore, Billerica, MA, USA). Specific proteins were detected using specific primary antibodies and horseradish peroxidase-conjugated secondary antibodies, together with enhanced chemiluminescence developing agent (Millipore). Images were digitally acquired with a Kodak Image Station 4000 (Carestream Health Inc., Rochester, NY, USA) or ImageQuant LAS 4000mini (GE, Uppsala, Sweden), and the companion software. The density of LC3-II was first normalized to the loading control, tubulin. Then, the ratio of LC3-II/tubulin is converted to numbers relative to that of the control treatment.

### Immunofluorescence staining

Cells were grown on 3 cm Petri dish and were fixed in 4% paraformaldehyde for 15 min. Cells were washed two times in phosphate-buffered saline (PBS), permeabilized with 0.1% Triton X-100 for 15 min, followed by another wash in PBS and block in PBS containing 5% bovine serum albumin (BSA) for 1 h. Primary and secondary antibodies in PBS containing 5% BSA were applied overnight at 4 °C and 1 h at room temperature, respectively. Nuclei were costained with 1 *μ*g/ml Hoechst 33342. Fluorescence images were taken using a Nikon Eclipse TE200 epifluorescence microscope with NIS-Elements AR3.2 software or EVOS FL Auto (Life Technologies, Bothell, WA, USA). For manual quantification of the puncta formation at least three optical fields with at least 50 cells per experimental condition were analyzed. Data from repeated experiments were subjected to statistical analysis.

### Electron microscopy

Electron microscopy was performed as described.^42^ Briefly, cells in 6-well plates were fixed in 2.5% glutaraldehyde (Sigma) in 0.1 M phosphate buffer (pH 7.4) for 2 h, and then dehydrated in a graded ethanol series and embedded. Ultrathin sections were mounted on copper grids. The samples were then stained and visualized using a 120 kV Jeol electron microscope (JEM-1400, Peabody, WA, USA) at 80 kV. Images were captured using a Gatan-832 digital camera (Pleasanton, CA, USA).

### Statistics

All experiments were performed at least in triplicate and data are presented as mean±S.D. The statistical significance of data was verified by the Student's *t*-test wherever required. *P*-value <0.05 was considered as being significant.

## Abbreviations

Atgs: autophagy genes
ATGs: autophagy proteins
Baf: bafilomycin A_1_
BFA: brefeldin A
ConA: concanamycin A
CQ: chloroquine
DFCP1: double FYVE-containing protein 1
GCA: golgicide A
LAP: LC3-associated phagocytosis
3-MA: 3-methyladenine
NCA: noncanonical autophagy
PBS: phosphate-buffered saline
PI3KC3: class III phosphatidylinositol-3 kinase
SalA: salicylihalamide A
ULK1: unc-51-like kinase 1
WIPI2: phosphoinositide-interacting protein 2 with WD repeat domain
WM: wortmannin
