# Supplementary material for: Golgi-associated LC3 lipidation requires V-ATPase in noncanonical autophagy
Source: Cell Death Dis. 2016 Aug 11;7(8):e2330–. doi: 10.1038/cddis.2016.236 (PMC5108321; doi:10.1038/cddis.2016.236)
Supplement: Supplementary Information [file cddis2016236x1.doc]

566-575.

**Supplemental data**

**Figure S1 (Supplemental to Fig. 1). AMDE-1-induced autophagy requires the ubiquitin-like conjugation system**

Atg5 KO-MEFs (A), Atg7KO-MEFs (B), Atg4bKO-MEFs (C) and Atg3KO-MEFs (D) were treated with or without AMDE-1 (10 μM) for 6 hours, and then analyzed by immuno-blotting for LC3-II formation.

**Figure S2 (Supplemental to Fig. 3). DFCP1 puncta formation requires FIP200 in AMDE-1-induced autophagy**

(A) WT-MEFs expressing GFP-DFCP1 were treated with or without AMDE-1 (10 μM) or rapamycin (Rap, 1 μM) for 6 hours and then assessed for LC3 staining (Green), DFCP1 puncta formation (Red), and Hoescht33342 staining (Blue). Arrows indicate examples of colocalized GFP-DFCP1 and LC3 signals. Bar= 25 μm. (B) FIP200KO-MEFs expressing GFP-DFCP1 were treated with or without AMDE-1 (10 μM) or rapamycin (Rap, 1 μM) for 6 hours and then assessed for LC3 staining (Green), DFCP1 puncta formation (Red), and Hoescht33342 staining (Blue). Bar= 25 μm.

**Figure S3 (Supplemental to Fig. 3). AMDE-1 does not affect Atg5-Atg12 conjugates in wild type and FIP200-deficient cells**

Immunoblotting analysis of ATG5-ATG12 conjugates in WT and FIP200KO-MEF after AMDE-1 treatments for 6 hours.

**Figure S4 (Supplemental to Fig. 4). AMDE-1-induced non-canonical autophagy recruits SQSTM1, but SQSTM1 does not affect LC3 lipidation**

(A-B) Hela cells expressing GFP-LC3 were treated by AMDE-1 (10 μM) and 3MA (10 mM) for 6 hours with or without SQSTM1-siRNA for 48h, followed by staining of SQSTM1 and LC3 (A) or by western blotting analysis (B). Arrows indicate examples of colocalizedGFP-LC3 and SQSTM1 signals. Bar= 25 μm.

**Figure S5 (Supplemental to Fig. 7). CQ does not affect AMDE-1-induced non-canonical autophagy**

FIP200KO-MEFs were treated by AMDE-1 (10 μM) with or without CQ (40 μM) for 6 hours followed by immunostaining for LC3 and GM130. Arrows indicate colocalization of LC3 with GM130 (D). Bar= 25 μm.

**Figure S6 (Supplemental to Fig. 7). Electron microscopic analysis of FIP200KO-MEFs treated with AMDE-1**

FIP200KO-MEFs were treated with AMDE-1 (10 μM) for 6 hours. Cells were analyzed by transmission electron microscopy. N= nuclei, AV=autophagosome, AL= autolysosome, LE=late endosome, M=mitochondria.
