# Supplementary figures and images for: Golgi-associated LC3 lipidation requires V-ATPase in noncanonical autophagy
Source: Cell Death Dis. 2016 Aug 11;7(8):e2330–. doi: 10.1038/cddis.2016.236 (PMC5108321; doi:10.1038/cddis.2016.236)

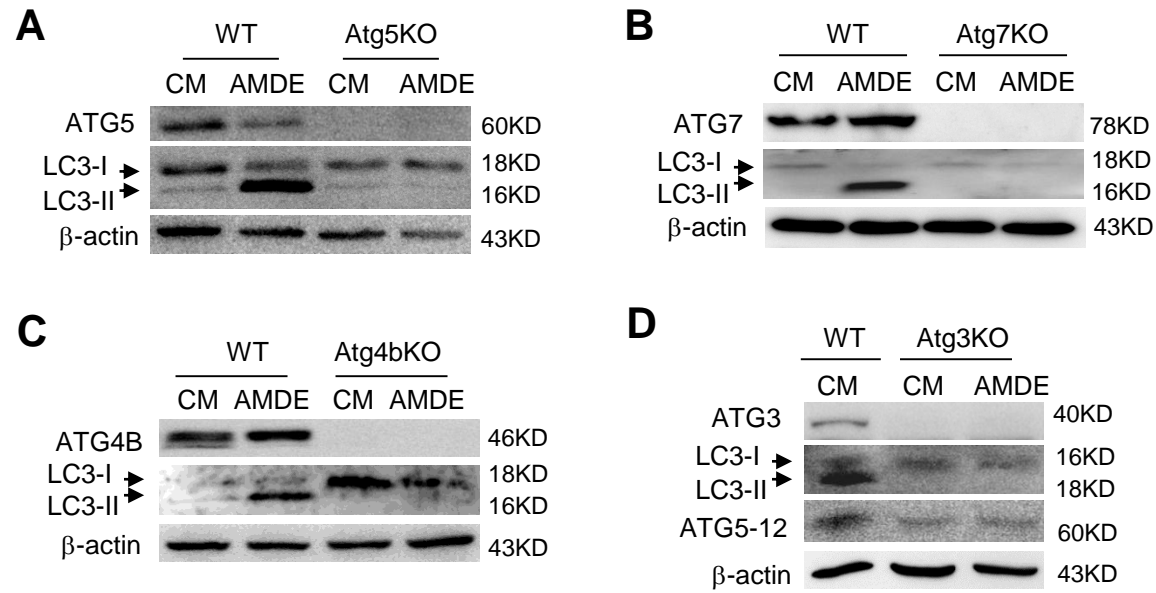

**A**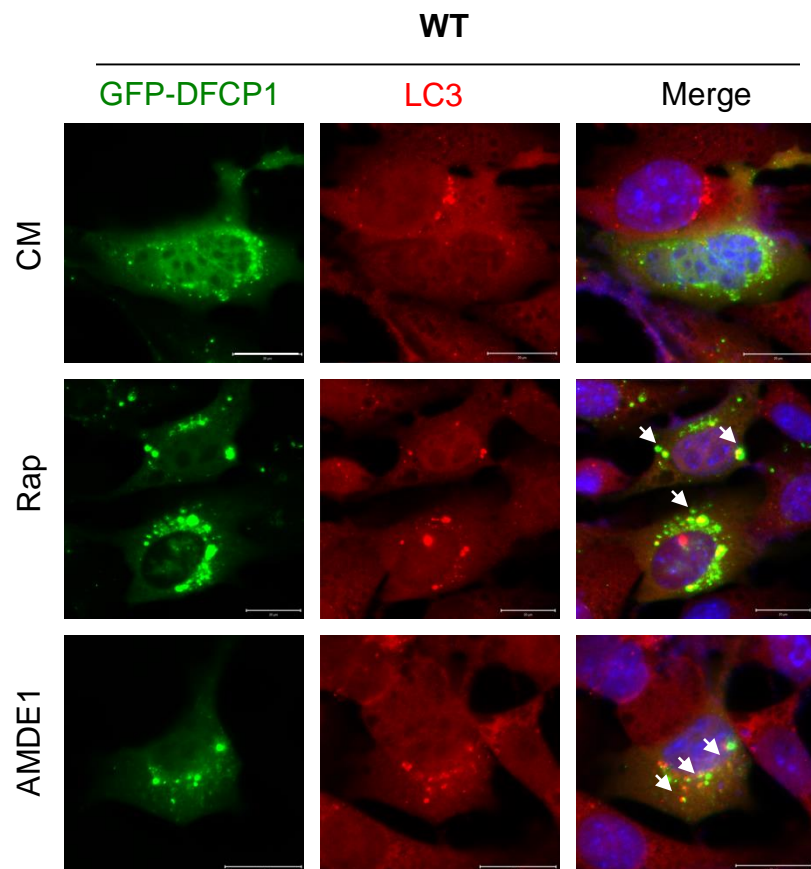**B**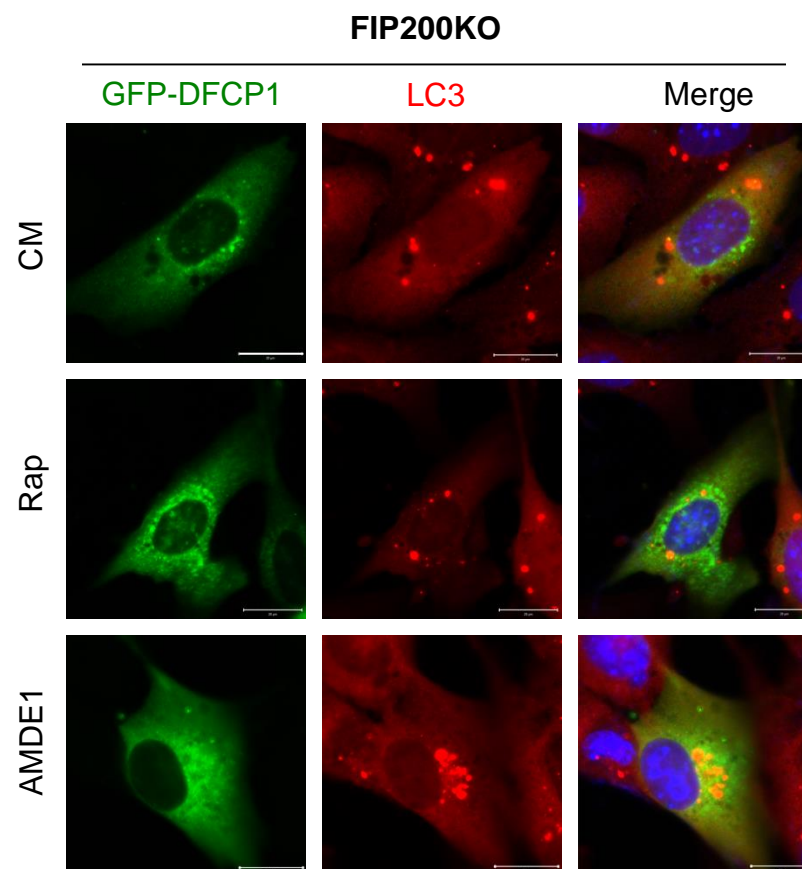

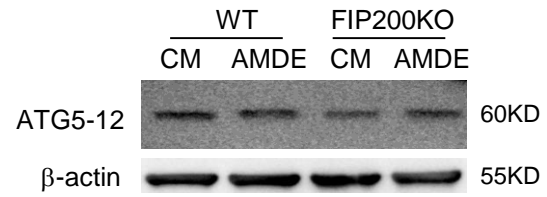

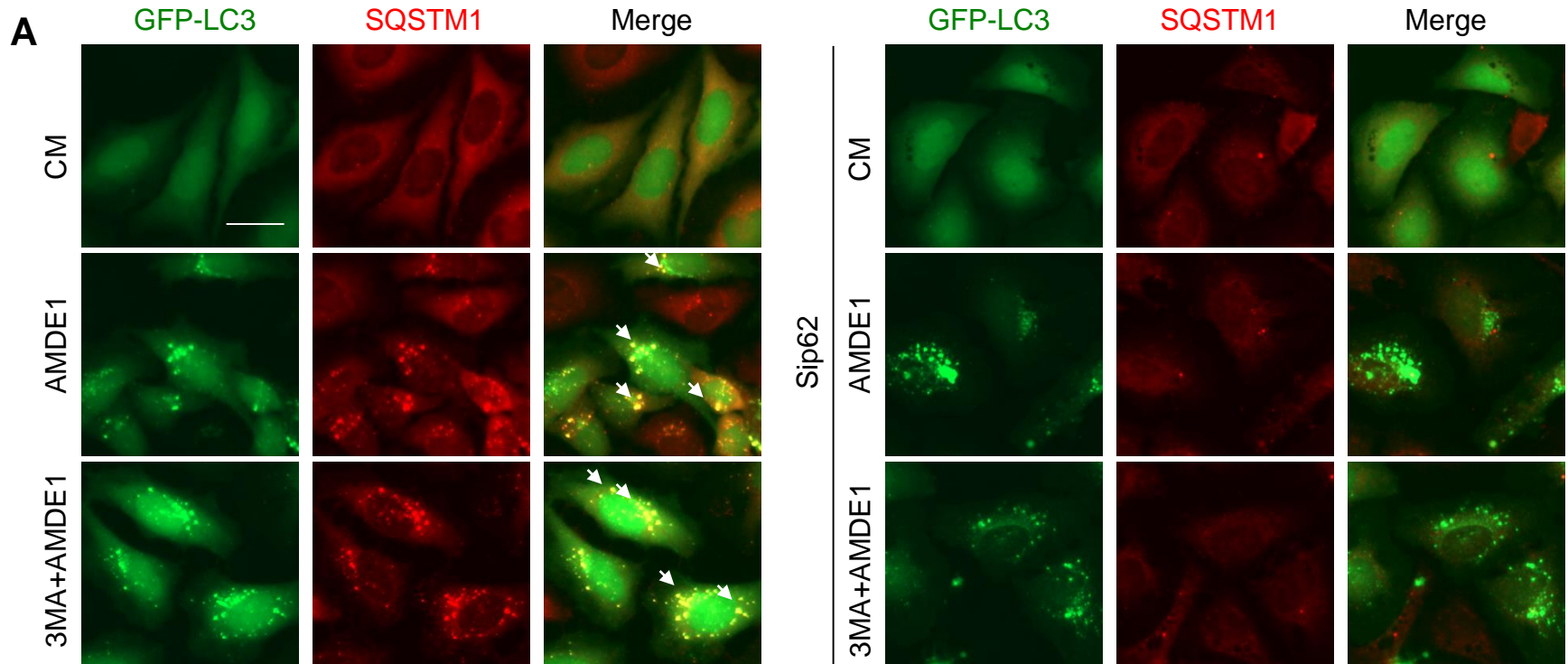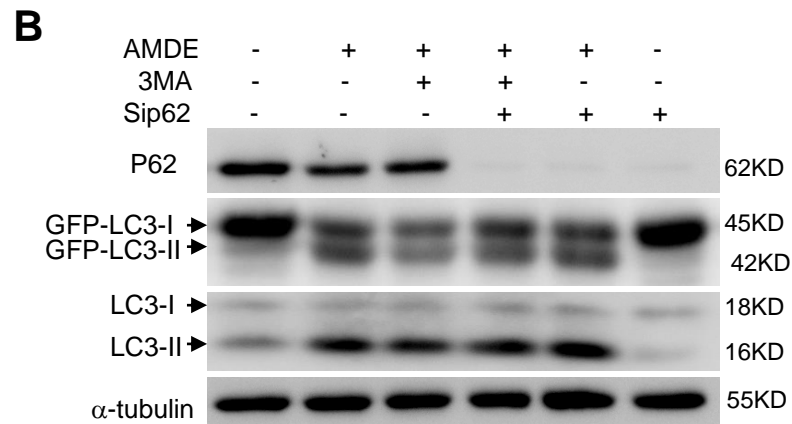

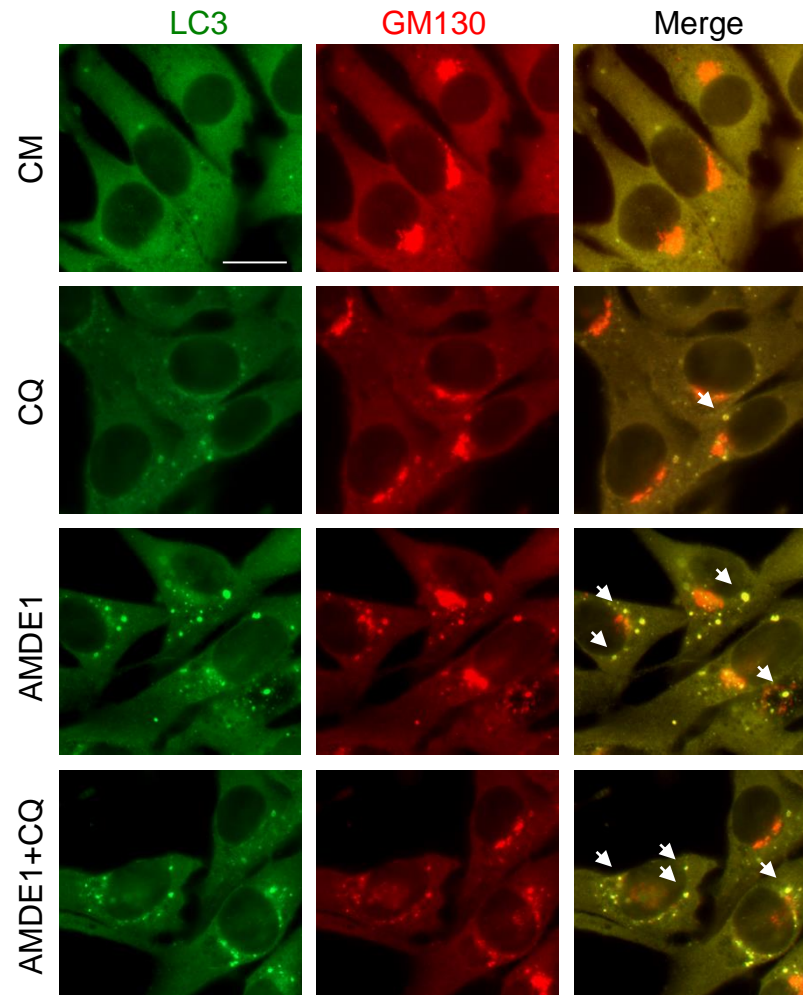

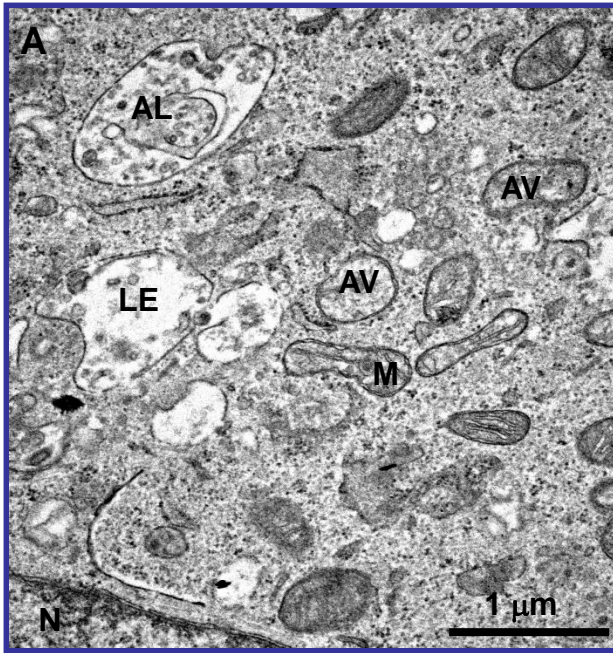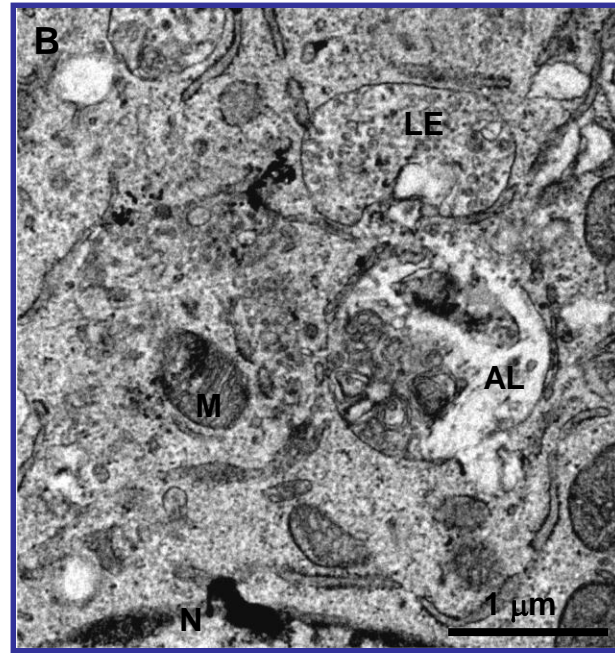

Supplement: Supplementary Figures [file cddis2016236x2.pdf]
